# Supplementary material for: Phase-based coordination of hippocampal and neocortical oscillations during human sleep
Source: Commun Biol. 2020 Apr 20;3:176. doi: 10.1038/s42003-020-0913-5 (PMC7170909; doi:10.1038/s42003-020-0913-5)
Supplement: Supplementary file 2 — Reporting Summary [file 42003_2020_913_MOESM2_ESM.pdf]

nature research

Corresponding author(s): Roy Cox

Last updated by author(s): Mar 23, 2020

Reporting Summary

Nature Research wishes to improve the reproducibility of the work that we publish. This form provides structure for consistency and transparency in reporting. For further information on Nature Research policies, see Authors & Referees and the Editorial Policy Checklist.

Statistics

For all statistical analyses, confirm that the following items are present in the figure legend, table legend, main text, or Methods section.

n/a	Confirmed	
☐	☒	The exact sample size (n) for each experimental group/condition, given as a discrete number and unit of measurement
☐	☒	A statement on whether measurements were taken from distinct samples or whether the same sample was measured repeatedly
☐	☒	The statistical test(s) used AND whether they are one- or two-sided<br>*Only common tests should be described solely by name; describe more complex techniques in the Methods section.*
☒	☐	A description of all covariates tested
☐	☒	A description of any assumptions or corrections, such as tests of normality and adjustment for multiple comparisons
☐	☒	A full description of the statistical parameters including central tendency (e.g. means) or other basic estimates (e.g. regression coefficient) AND variation (e.g. standard deviation) or associated estimates of uncertainty (e.g. confidence intervals)
☐	☒	For null hypothesis testing, the test statistic (e.g. F, t, r) with confidence intervals, effect sizes, degrees of freedom and P value noted<br>*Give P values as exact values whenever suitable.*
☒	☐	For Bayesian analysis, information on the choice of priors and Markov chain Monte Carlo settings
☒	☐	For hierarchical and complex designs, identification of the appropriate level for tests and full reporting of outcomes
☒	☐	Estimates of effect sizes (e.g. Cohen's d, Pearson's r), indicating how they were calculated

Our web collection on statistics for biologists contains articles on many of the points above.

Software and code

Policy information about availability of computer code

Data collection	Electrophysiological data were recorded using a Stellate recording system (Stellate GmbH, Munich, Germany).
Data analysis	Data were analyzed with Matlab (R2018a), using custom code in conjunction with several freely available toolboxes (EEGlab, CircStat).

For manuscripts utilizing custom algorithms or software that are central to the research but not yet described in published literature, software must be made available to editors/reviewers. We strongly encourage code deposition in a community repository (e.g. GitHub). See the Nature Research guidelines for submitting code & software for further information.

Data

Policy information about availability of data

All manuscripts must include a data availability statement. This statement should provide the following information, where applicable:

- Accession codes, unique identifiers, or web links for publicly available datasets
- A list of figures that have associated raw data
- A description of any restrictions on data availability

Data are not publicly available due to privacy concerns related to clinical data, but data are available from the corresponding or senior author upon obtaining ethical approval.

Field-specific reporting

Please select the one below that is the best fit for your research. If you are not sure, read the appropriate sections before making your selection.

☒ Life sciences ☐ Behavioural & social sciences ☐ Ecological, evolutionary & environmental sciences

Life sciences study design

All studies must disclose on these points even when the disclosure is negative.

Sample size	No sample size calculation was performed. Data were taken from archival dataset and included when patients had >10 min of clean data per sleep stage following artifact rejection, and electrodes implanted in non-pathological hippocampus and lateral temporal cortex.
Data exclusions	All group analyses are based on the full sample (N=10), except for one analysis of N=8 (shown in Figure 5) due to insufficient data to generate surrogate distributions.
Replication	In addition to group-level analyses, single-subject analyses indicated the presence of group effects across multiple subjects (Supplementary Figures 3, 6, 8).
Randomization	Randomization not relevant for one group.
Blinding	Blinding not relevant for one group.

Reporting for specific materials, systems and methods

We require information from authors about some types of materials, experimental systems and methods used in many studies. Here, indicate whether each material, system or method listed is relevant to your study. If you are not sure if a list item applies to your research, read the appropriate section before selecting a response.

Materials & experimental systems

n/a	Involved in the study	
☒	☐	Antibodies
☒	☐	Eukaryotic cell lines
☒	☐	Palaeontology
☒	☐	Animals and other organisms
☐	☒	Human research participants
☒	☐	Clinical data

Methods

n/a	Involved in the study	
☒	☐	ChIP-seq
☒	☐	Flow cytometry
☒	☐	MRI-based neuroimaging

Human research participants

Policy information about studies involving human research participants

Population characteristics	Patients suffering from pharmaco-resistant epilepsy (age: 36.6 ± 14.8 yrs, range: 22–62) with 22.5 ± 11.0 yrs (range: 10–49) disease history.
Recruitment	See section "Sample size" above.
Ethics oversight	Ethics committee of the Medical Faculty of the University of Bonn.

Note that full information on the approval of the study protocol must also be provided in the manuscript.
